# From Synthesis Optimization to Chelation Mechanism: A Rice Protein Peptide–Calcium Complex Enhances Intestinal Calcium Absorption and Bone Formation via the TRPV6-Calbindin9k Axis

**DOI:** 10.3390/foods15142490

**Published:** 2026-07-14

**Authors:** Yue Tian, Wenting Yang, Yangzheng He, Xin Bi, Yong Sun

**Affiliations:** 1 State Key Laboratory of Food Science and Resources, Nanchang University, Nanchang 330047, China; 407900230061@email.ncu.edu.cn (Y.T.); wentyyang1011@126.com (W.Y.); 357900220006@email.ncu.edu.cn (Y.H.); 2 Jiangxi Medicine Academy of Nutrition and Health Management, Nanchang University, Nanchang 330006, China

**Keywords:** rice protein peptide–calcium chelate, chelating mechanism, calcium absorption-promoting effect, osteoporosis, TRPV6, Calbindin-D9k

## Abstract

Rice protein peptides, abundant byproducts of rice processing, represent a sustainable source for developing novel nutritional delivery systems. To address the low bioavailability of traditional calcium supplements, this study aimed to fabricate a high-performance calcium-chelating complex (RPP-Ca) and elucidate its functional mechanism. The synthesis process was systematically optimized, yielding a maximum calcium-binding capacity of 93.98 ± 1.99 mg/g under optimal conditions (pH 10, 70 °C, 50 min reaction time, peptide-to-calcium mass ratio of 2:1). Physicochemical characterization utilizing scanning electron microscopy (SEM) and Fourier transform infrared spectroscopy (FTIR) confirmed successful chelation, revealing significant microstructural reorganization and enhanced thermal stability compared to native peptides. Functional validation via in vitro Caco-2 cell models and in vivo calcium-deficient mouse models demonstrated that RPP-Ca significantly promotes intestinal calcium absorption and osteogenesis. Mechanistically, these effects were mediated through the activation of the TRPV6-Calbindin9k signaling axis. These findings underscore the potential of industrial rice protein peptides as an effective and bioavailable calcium fortification ingredient, providing a theoretical basis for the high-value utilization of rice byproducts in functional foods.

## 1. Introduction

Rice is widely grown in Asia, Africa, the Americas, and Europe and is the staple food for more than half the world’s population [1]. Rice protein (RP) is the primary protein fraction native to rice, typically isolated by removing starch via enzymatic hydrolysis. It possesses a well-balanced amino acid profile, high biological value, superior protein utilization, low allergenicity and antigenicity, as well as high digestibility; its content in rice is second only to starch [2,3]. About 15% of the broken rice produced during rice processing, although the composition is the same, is only 30–50% of the value of rice and is usually used as feed [4]. After more than 10 years of technical research, our team has cooperated with Jiangxi Hengding Food Co., Ltd. (a national high-tech enterprise) to develop a product chain that uses broken rice as raw material to produce rice high fructose syrup, rice oligosaccharides, special microcapsule powder oil, rice protein and rice protein peptide (RPP), realizing the full price utilization of rice resources. The RP and RPP have been mass-produced and exported, with sales of more than 3600 tons in 2021 and an output value exceeding $3 million. With a molecular weight below 2500 Da, this industrially produced RPP is a low molecular weight peptide [5], which may have high calcium binding capacity [6].

Recently, various enzymes have been used to improve the physical and chemical properties, functions, and immune properties of natural proteins to increase protein bioavailability and obtain protein peptides with high selectivity and fewer toxic side effects [7,8]. As a new food resource, RPP has attracted more and more attention because of its various physiological activities. The peptides isolated from rice endosperm protein hydrolysate showed good antioxidant activity [9]. The study also found that rice peptides can prevent hyperuricemia and kidney damage [10]. Our previous studies also demonstrated that RP and RPP could alleviate dextran sulfate sodium (DSS)-induced acute colitis in mice through the Keap1-Nrf2 signaling pathway and the modulation of gut microbiota [3,5].

Calcium, which accounts for 1.5% to 2.2% of total body weight, is the most common mineral in the human body [11]. It is involved in diverse biological activities in the body, such as maintaining the permeability of cell membranes and capillaries, metabolism within cells, nerve conduction, muscle contraction, promotion of enzyme activity and blood coagulation [12]. A statistical result of the calcium intake of adults in 74 countries and regions in the world showed that the countries with an average daily calcium intake of more than 1000 mg/d were all Nordic countries, and 400–600 mg/d in South America, while in Asian countries including China, it was less than 400 mg/d [13], which was far lower than the recommended calcium intake of Chinese nationals (1200 mg/d). Insufficient calcium intake can cause diseases including rickets, osteoporosis, kidney stones, and arterial hypertension [14]. Calcium deficiency in most areas is caused by insufficient dietary intake, and proper calcium supplementation is an effective way to maintain health. At now, numerous varieties of dietary calcium supplements are available, including inorganic calcium, amino acid calcium chelates, and peptide calcium chelates. However, compared with peptide–calcium chelates, other calcium supplements have many disadvantages or side effects, such as gastrointestinal irritation, low bioavailability, and sensitivity to lipid oxidation [15,16,17]. Compared with amino acids, bioactive peptides are effective calcium transporters with good absorption, low energy requirements and high loading capacity [18]. A wheat germ polypeptide with the amino acid sequence Phe-Val-Asp-Val-Thr showed 89.94% calcium ion chelation ability [19]. Phe-Tyr peptide isolated and purified from seaweed had a Ca^2+^ binding ability of 128.77 μg/mg; this peptide–calcium chelate exhibited high thermal stability and solubility, which facilitates its uptake and transport in the intestinal tract [14]. Peptide-calcium chelates prepared from tilapia can promote bone formation by increasing calcium bioavailability and bone collagen content [20]. Although various biopeptides are already used to produce peptide–calcium chelates, they have not yet been industrially produced and applied in large quantities. In addition, few studies about RPP-Cas have been reported. It is worth noting that the specific active peptide sequence responsible for calcium chelation, identified as AHVGMSGEEPE, has been previously characterized by our group [21]. While our previous work elucidated the molecular binding mechanism of this specific sequence, the present study focuses on evaluating the macroscopic functional properties and overall efficacy of the unseparated peptide–calcium complex as an integrated raw material, laying the groundwork for its future functional fractionation and commercial application.

In this study, the peptide calcium chelates were synthesized using industrially produced RPP, and their calcium absorption-promoting effect and mechanism were investigated using a Caco-2 monolayer cell model. In addition, we conducted animal experiments to compare the effects of RPP-Ca, CaCl_2_ and chelated RPP-Ca on the absorption of calcium. The results of this study not only expand RPP’s application range but also enrich the variety of calcium supplements.

## 2. Materials and Methods

### 2.1. Materials and Reagents

RPP was provided by Jiangxi Hengding Food Co., Ltd. (Jiangxi, China). Caco-2 cells were procured from the Shanghai Institute of Biological Sciences, Chinese Academy of Sciences. Fluorescein sodium salt (FSS) and MTT were obtained from Aladdin (Shanghai, China). FBS was obtained from Biological Industries biotech company (Israel). RNAeasy^TM^ Animal RNA Extraction Kit was obtained from Beyotime Biotechnology (Shanghai, China). PBS, HBSS (calcium-free), and DMEM were obtained from Solarbio (Beijing, China). The real-time polymerase chain reaction (PCR) kits were obtained from TaKaRa (Shiga, Japan). The test kits for alkaline phosphatase (ALP), calcium content, and phosphorus content were purchased from Nanjing Jiancheng Bioengineering Institute (Nanjing, China). The ELISA kit for osteocalcin (OCN) was purchased from Shanghai Bohu Biotechnology Co., Ltd. (Shanghai, China).

### 2.2. Chemical and Physical Characterization of the RPP-Ca

#### 2.2.1. Preparation of RPP-Ca

**Single-factor experiment.** The influence of peptide weight ratio, calcium, pH, temperature and time on calcium chelating rate was investigated by one-way experiments. Briefly, rice protein peptide (RPP) was dissolved in 10 mL deionized water, then 50 mg CaCl_2_ was added to prepare the peptide–calcium solution with the weight ratio of peptide: calcium (1:1, 2:1, 3:1, 4:1 and 5:1). After fully dissolving, vortexing and mixing, the chelating reaction was carried out with pH (6, 7, 8, 9 and 10), temperature (30, 40, 50, 60 and 70 °C) and time (30, 40, 50, 60 and 70 min). After the reaction is finished and has cooled to room temperature, add three times the volume of anhydrous ethanol and let stand for 60 min. Following centrifugation at 8000 *g* for 15 min, the precipitate was harvested, washed thrice with anhydrous ethanol, and subsequently freeze-dried to produce RPP-Ca. Only one factor changed in each experiment, and the other factors remained unchanged. The influence of four factors was investigated successively.

**Orthogonal optimization experiment.** Employing single-factor test outcomes, orthogonal optimization was performed using a three-level, four-factor design (peptide-to-calcium ratio, pH, temperature, and time) to improve the process of preparing RPP-Ca. The experimental design is illustrated in Table 1.

#### 2.2.2. Calcium Binding Capacity Analysis

The calcium concentration in RPP-Ca chelate was quantified according to the National Standards of China (GB 5009.92-2016) [22] by a flame atomic absorption spectrophotometer (PinAAcle 900F, PerkinElmer, Waltham, MA, USA). Calcium binding capacity was expressed as calcium content in the chelate (mg/g chelate).

#### 2.2.3. Scanning Electron Microscope (SEM) Analysis

The microstructure of RPP and RPP-Ca was detected with SEM (JSM-6701F, JEOL, Tokyo, Japan) according to our previous methods [5]. Briefly, the dried samples were uniformly coated with conductive adhesive, and then the samples were sprayed with gold. The morphology and structure of each sample were observed using an SEM at an accelerating voltage of 5 kV.

#### 2.2.4. DSC Analysis

The DSC curve was obtained using the differential scanning calorimeter (DSC 8000, PerkinElmer, USA). The temperature ranged from 25 to 200 °C at a rate of 10 °C/min, with a nitrogen flow rate of 20 mL/min.

#### 2.2.5. Thermal Gravimetric Analyzer (TGA)

RPP and RPP-Ca samples were placed in a crucible and determined by a thermogravimetric analyzer (TGA 4000, PerkinElmer, USA). The measured temperature was 30–800 °C (10 °C/min), and the nitrogen flow rate was 20 mL/min. The real-time weight of the samples was documented, and the mass loss rate waveform for each sample was computed and illustrated.

#### 2.2.6. Amino Acid Composition

The amino acid composition in RPP-Ca was quantified utilizing an automated amino acid analyzer (S-433D, Sykam, Munich, Germany) [5,22]. In summary, 25 mg samples were precisely measured and introduced into a 20 mL ampoule, followed by the addition of 10 mL of 6 M HCl solution and 1 mL of phenol. Subsequent to the nitrogen depletion, the tube underwent hydrolysis in an oven at 110 °C for 24 h. The hydrolyzed sample was analyzed using an automated amino acid analyzer.

#### 2.2.7. Ultraviolet-Visible Spectroscopy (UV-Vis) Analysis

RPP and RPP-Ca were dissolved in ultrapure water to prepare a 0.05 mg/mL solution. The ultrapure water was used as the reference. The UV-vis spectrum of each sample was scanned in the wavelength range of 190–350 nm with a UV-vis spectrophotometer (TU-1900, PERSEE, Beijing, China).

#### 2.2.8. Fourier Transform Infrared Spectrometer (FTIR) Analysis

RPP and RPP-Ca (1 mg) were combined with KBr (100 mg) and subsequently ground thoroughly in an agate mortar to produce translucent thin sections under a pressure of 30 MPa. The infrared absorption spectra of each sample in 4000–400 cm^−1^ were recorded by an infrared spectrometer (Spectrum Two, PerkinElmer, USA).

### 2.3. Evaluation of Promoting Calcium Absorption In Vitro

#### 2.3.1. Cell Culture and Viability Assay

Caco-2 cells were cultivated at 37 °C with 5% CO2 in DMEM mixed with 10% (*v*/*v*) FBS and 1% penicillin/streptomycin. Cell viability was assessed using the MTT test in accordance with our established protocols [23].

#### 2.3.2. Caco-2 Cell Monolayer Model

The Caco-2 cell monolayer model was constructed according to our prior methodology [23]. Cells were inoculated at a density of 3 × 10^5^ cells/mL into the apical chamber (1.5 mL) of a Transwell plate (24 mm in diameter, produced by Corning), while the basolateral chamber was subsequently filled with 2.6 mL of culture media. Beginning on the third day following inoculation, the transepithelial electrical resistance (TEER) values of each well were assessed before medium replacement every two days, and the culture was sustained for 21 days. TEER was assessed and computed according to our prior methodology [23].

#### 2.3.3. Apparent Permeability Coefficient (Papp)

The permeability of the Caco-2 cell monolayer membrane was assessed using fluorescein sodium salt. Following the washing of Caco-2 cells cultivated for 21 days with HBSS buffer three times, 1.5 mL of fluorescein sodium salt solution produced in HBSS was given into the apical chamber, while 2.6 mL of HBSS solution was injected into the basolateral chamber. Samples were collected from the basolateral chamber at 0.5, 1, 1.5, and 2 h, and an equivalent volume of HBSS solution was added. The fluorescence intensity of each sample was detected, and the concentration of fluorescein sodium salt in HBSS in the basolateral chamber was calculated using the standard curve. Papp was calculated based on our previous method [24].

#### 2.3.4. Calcium Transport Assay

The Caco-2 cell monolayer was rinsed thrice with HBSS buffer, followed by the addition of 0.5 mL HBSS buffer containing varying concentrations of RPP-Ca (2, 4, 6 mg/mL) or CaCl_2_ (1.69 mM, corresponding to the calcium content in 2 mg/mL RPP-Ca) to the apical chamber, while 1.5 mL HBSS solution was introduced to the basolateral chamber. 0.5 mL samples were extracted from the basolateral chamber at 0.5, 1, 1.5, and 2 h, and an equivalent volume of HBSS solution was subsequently added. Post-experiment, all Caco-2 cells were harvested for further PCR analysis. The calcium concentration of the collected samples was ascertained via atomic absorption, and the transported calcium concentration of each well was computed using the following formula:

(1) $$B_{n}\;=\;1.5\;\times \;A_{n}\;+\;0.5\;\times \;\sum_{k=1}^{n-1}A_{k}$$

where B_n_ is the transported calcium content in the basolateral chamber of each well at different times; A_n_ is the calcium content of the collected samples at different times; n, an independent variable, is 1, 2, 3, and 4, representing the sampling time points of 0.5, 1, 1.5, and 2 h, respectively.

#### 2.3.5. Real-Time PCR

Total RNA was extracted from the collected Caco-2 cells with the RNAeasy^TM^ Animal RNA Extraction Kit, and its purity and concentration were determined. The RNA was reverse transcribed into cDNA according to the instructions provided by the reverse transcription kit. The fluorescent quantitative reaction was conducted in accordance with the PCR kit. The reaction parameters were pre-denaturation at 95 °C for 30 s, 95 °C for 5 s, 56.5 °C for 30 s, PCR for 39 cycles, 60 °C for 5 s, and 95 °C for 50 s. The primer sequences of each gene are shown in Table S1.

### 2.4. Evaluation of Promoting Calcium Absorption and Bone Synthesis In Vivo

#### 2.4.1. Animal Experiment Design

Forty healthy KM mice (male, 4 weeks old, 25 ± 5 g) were acquired from Home-SPF (Beijing) Biotechnology Co., Ltd. (Certification number: SCXK2024–0001). All experimental protocols received permission from the Animal Ethics Committee of Jiangxi University of Traditional Chinese Medicine (Acceptance number: 20250118002). Throughout the experimental duration, mice were provided unrestricted access to deionized water and food, housed in stainless steel cages with regulated indoor temperature (21 ± 1 °C) and humidity (60 ± 10%), and maintained on a 12 h light/dark cycle. Following one week of acclimatization, the mice were randomly assigned to groups.

During the initial four weeks, the experimental group was administered a calcium-deficient diet, thus establishing the calcium deficit model. Subsequently, during the ensuing four weeks, the experimental group received the medicine according to the specified design:

1. The CON group: Normal calcium diet group (0.5% Ca) gavaged with equal volumes of saline;
2. The MOD group: The calcium-free diet group gavaged with equal volumes of saline;
3. The CaCl_2_ group: The calcium-free diet group gavaged with CaCl_2_;
4. The RPP-Ca group: The calcium-free model group gavaged with RPP-Ca, which is made by RPP chelated with CaCl_2_;
5. The RPP + Ca group: The calcium-free model group gavaged with RPP and CaCl_2_, which is called RPP + Ca in the following sections.

The intragastric dosage was 1 mL/100 g body weight, and the daily calcium content was 93.31 mg Ca/kg body weight, which is seven times the required daily consumption of 800 mg Ca per 60 kg body weight (13.33 mg Ca/kg body weight). Mice were anesthetized with isoflurane on the last day of intragastrication and euthanized after fasting for 9 h. Abdominal blood was collected, centrifuged at 2000 *g* at 4 °C for 15 min, and clarified serum was obtained. The small intestine and colon were preserved at −80 °C immediately upon collection. The femur and tibia were separated, muscle and soft tissues were excised, and rinsed thrice with saline. The left femur and tibia were encased in saline-soaked gauze, thereafter coated with aluminum foil and preserved at −20 °C. The left femur and tibia were encased in 4% paraformaldehyde and preserved using four sheets of aluminum foil. The right femur was preserved in 4% paraformaldehyde and wrapped in aluminum foil. Organs, including kidneys, spleens, and livers, were harvested and weighed. The organ index is computed as follows:

(2) $$\mathrm{organ}\;\mathrm{index}\;(g/g)\;=\;\frac{\mathrm{weight}\;\mathrm{of}\;\mathrm{organ}\;(g)}{\mathrm{body}\;\mathrm{weight}\;(g)}$$

#### 2.4.2. Measurement of Serum Parameters

ALP content was measured utilizing the appropriate commercial kit from Nanjing Jiancheng Bioengineering Institute, Nanjing, China; OCN levels were assessed using an ELISA kit from Pyram, Shanghai, China, in accordance with the provided instructions.

#### 2.4.3. Bone Biomechanics

MultiTest 2.5-i (Mecmesin, Horsham, UK) was used to perform three-point flexural mechanics tests on each right femoral shaft. The right femur of the rat was extracted at -20 °C, thawed at ambient temperature for 2 h, and subsequently positioned in a texture analyzer. The femur was fixed on a platform with a span of 12 mm (15 mm for the tibia test) and measured using an HDP/3PB probe in TA.XT compression mode. The loading speed of the probe is 10 mm/min, and the operating height is 3 cm. The load deformation chart was recorded, and the diameter of the external axis (mm), external minor axis (mm), internal major axis (mm) and internal minor axis (mm) were measured with a vernier caliper. The moment of inertia (J) of the bone cross-section is calculated, and the maximum stress σ_max_ (N/mm^2^) is obtained. The calculation method is as follows:

(3) $$J\;(mm^{4})\;=\;\frac{\pi (BH^{3}-bh^{3})}{64}$$

(4) $$\sigma_{\max}\;(N/mm^{2})=\frac{9.8\times 10^{-3}\times \left(10\mathrm{LH}\right)}{8\;J}$$

where L is the maximum load (g); B is the external axis diameter of bone (mm); H is the external short axis diameter (mm); b is the inner major axis diameter (mm); h is the inner short axis diameter (mm); and $\sigma_{\max}$ represents the maximum stress (N/mm^2^).

Following testing, the broken femur and tibia were desiccated in an oven at 110 °C until a consistent weight was achieved and documented, after which the bone weight index was computed:

(5) $$\mathrm{Bone}\;\mathrm{Weight}\;\mathrm{Index}\;(10^{-3})\;=\;\frac{\mathrm{Dried}\;\mathrm{Bone}\;\mathrm{Weight}}{\mathrm{Mouse}\;\mathrm{weight}}\times 1000$$

#### 2.4.4. Micro-Computed Tomography (CT) Analysis

The sample was removed from 4% paraformaldehyde, the excess liquid was dried with gauze, the sample was placed on the scanning bed of the instrument, and then scanning began. The scanning parameters were recorded in Table S2, and the original image was obtained after scanning.

#### 2.4.5. Histological Analysis of Bone

The left femur was fixed in a phosphate-formaldehyde buffer for 24 h, after which paraffin slices were stained with hematoxylin–eosin (H&E) and examined under a light microscope (Nikon Eclipse E100; Nikon, Tokyo, Japan) to assess the morphology of the mice’s femoral heads.

### 2.5. Statistical Analysis

Data were presented as mean ± standard deviation, with a significance threshold of *p* < 0.05, determined by one-way analysis of variance (ANOVA) followed by Dunnett’s test using SPSS (version 25.0, IBM, Armonk, NY, USA).

## 3. Results and Discussion

### 3.1. Optimization of the Synthesis Parameters of Calcium Chelates of RPP-Ca

#### 3.1.1. Effects of Different Synthesis Parameters on Calcium Binding Ability

**Figure 1:**
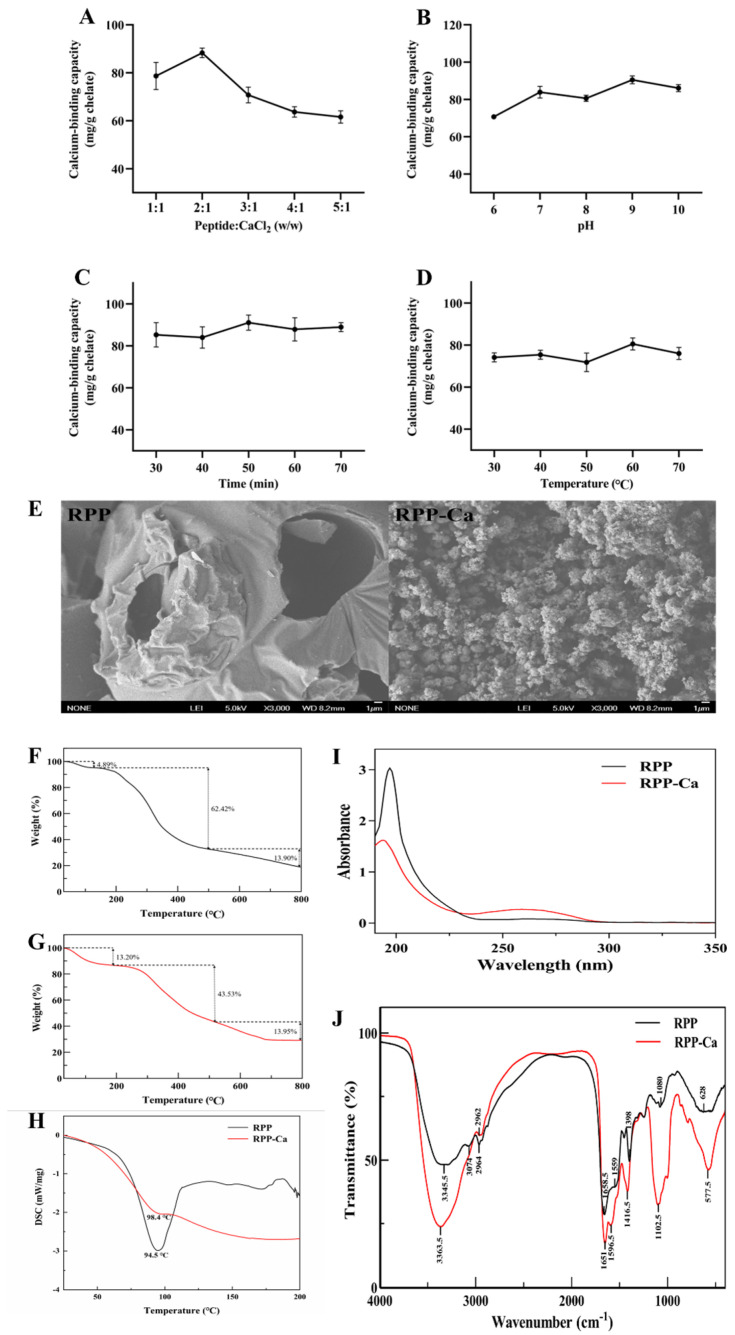
Effects of preparation conditions on calcium-binding capacity and structural characterization of RPP-Ca. (**A**) Effect of peptide to CaCl_2_ mass ratio (*w*/*w*); (**B**) Effect of pH; (**C**) Effect of chelation time; (**D**) Effect of reaction temperature on the calcium-binding capacity of RPP; (**E**) Scanning electron microscopy (SEM) images of RPP and RPP-Ca; Thermogravimetric analysis (TGA) curves of (**F**) RPP and (**G**) RPP-Ca; (**H**) Differential scanning calorimetry (DSC) thermograms of RPP and RPP-Ca; (**I**) Ultraviolet-visible (UV-vis) absorption spectra of RPP and RPR-Ca; (**J**) Fourier transform infrared (FTIR) spectra of RPP and RPP-Ca.

During the synthesis of RPP-Ca, we investigated the effects of the calcium peptide ratio, pH, reaction time, and temperature on the chelating ability between calcium ions and rice protein peptides (RPP). The calcium-binding capacity of the chelates first rose at pH 7, a reaction temperature of 50 °C, and a reaction duration of 50 min. However, when the peptide-to-calcium weight ratio increased, this capacity subsequently decreased (Figure 1A). This decrease might be explained by the saturation of calcium ions, which lowers the peptide’s usage efficiency when RPP is added continuously. At a 2:1 peptide-to-calcium ratio, RPP exhibited the greatest calcium chelating activity, with a binding capacity of 88.30 ± 1.42 mg/g. Consequently, peptide–calcium mass ratios of 1:1, 2:1, and 3:1 were selected for further orthogonal optimization experiments. Due to the competition between H^+^ and Ca^2+^ ions for the COO^−^ groups [25], the chelate’s calcium-binding ability was modest (70.70 ± 0.46 mg/g) at pH 6 under a 2:1 peptide-to-calcium ratio (Figure 1B). With an improvement in pH, the chelates’ ability to bind calcium increased, reaching a high of 90.47 ± 0.49 mg/g at pH 9. At pH levels above 10, Ca^2+^ ions precipitate as Ca(OH)_2_, which hinders chelation. Thus, pH values of 8, 9, and 10 were chosen for subsequent optimization experiments. At a 2:1 peptide–calcium ratio, a pH of 9, and a reaction time of 50 min, the chelate exhibited the strongest calcium-binding ability (91.10 ± 1.64 mg/g) when the reaction time was maintained at 50 min, while binding capacity gradually decreased with extended reaction times (Figure 1C). Consequently, reaction times of 40, 50, and 60 min were selected for further experiments. Finally, under the conditions of a 2:1 peptide–calcium ratio, 50 min of reaction time, and pH 9, the highest calcium-binding ability of the chelate was observed at 60 °C, with a binding capacity of 80.55 ± 2.04 mg/g (Figure 1D). The chelation of peptide with Ca^2+^ is an endothermic reaction, and the binding ability may be reduced by the carbonyl ammonia reaction when the temperature is too high [26]. Therefore, for the following orthogonal optimization experiments, the reaction temperatures of 50, 60, and 70 °C were chosen.

#### 3.1.2. Optimization of Peptide–Calcium Chelation Parameters

Building on the previous one-factor tests, we designed a three-level, four-factor orthogonal test to optimize the chelation parameters of calcium-chelated rice protein peptides (RPP-Ca). According to Table 2, the factors affecting RPP-Ca’s ability to chelate calcium were arranged as follows: B > C > A > D. The optimal preparation conditions for RPP-Ca were identified as B_3_C_2_A_2_D_3_. Under these optimal conditions, validation experiments demonstrated a calcium-binding capacity of 93.98 ± 1.99 mg/g of chelate, which exceeded the capacities observed in other experimental groups, thereby confirming the efficacy of this protocol. Overall, the optimized conditions for synthesizing RPP-Ca were determined to be pH 10, a reaction time of 50 min, a peptide-to-calcium mass ratio of 2:1, and a reaction temperature of 70 °C.

### 3.2. Structural Characterization of RPP-Ca

#### 3.2.1. SEM Observations Indicate the Formation of Crystals Between RPP and Calcium

We further employed scanning electron microscopy (SEM) to examine the surface microstructure of the synthesized RPP-Ca and compared it with that of the unchelated RPP. As shown in Figure 1E, after chelation with Ca^2+^, the surface of RPP transitioned from a smooth porous structure to a dense granular morphology. This alteration is likely owing to the breakdown of the original structure resulting from the coordination between RPP and Ca^2+^. Additionally, crystal-like structures were observed on the surface of RPP-Ca, which may represent calcium salt crystallites that have adhered to the RPP surface. A similar phenomenon was documented in a study on porcine collagen peptide calcium chelates [12]. The above results proved that a chelation reaction occurred between RPP and Ca^2+^.

#### 3.2.2. TGA and DSC Analysis Showed Enhanced Thermal Stability of RPPCa After Chelation

The thermogravimetric curves of the RPP and RPP-Ca from 30 °C to 800 °C are shown in Figure 1F,G, respectively, including three stages of weight loss. As the temperature increased from 30 to 133 °C, the weight loss of RPP was approximately 4.89% attributable to the volatilization of adsorbed water. As the temperature increased, the chemical bonds of RPP were disrupted at elevated temperatures, resulting in a weight loss of around 62.42% at 500 °C. During this stage, RPP released bound water and produced thermal degradation products. The weight loss of RPP was 13.90% at 800 °C, and the final residual weight was about 18.72%, mainly including the amino acid residue produced by the breaking of the peptide bond in RPP [27]. Compared with RPP, the weight loss of RPP-Ca was about 13.20%, 43.53%, and 13.95% at three stages, 30–190 °C, 190–520 °C and 520–800 °C, respectively. The final residual weight (29.17%) was higher than that of RPP, which was due to the metal oxides and amino acid residues in the residues of RPP-Ca [28]. The decomposition temperature of RPP-Ca was higher than that of RPP, possibly due to the existence of coordination bonds between RPP and Ca^2+^, which required higher temperatures to be destroyed [29]. The results showed that RPP-Ca had higher thermal stability compared with RPP, which may be due to the new chemical bond formation between RPP and calcium.

We also performed the DSC analysis to observe the thermal denaturation of RPPCa, As shown in Figure 1H of the DSC curve, the thermal absorption peak of RPP appeared at 94.5 °C, indicating that the thermal denaturation of RPP occurred at this temperature, while that of RPP-Ca appeared at 98.4 °C, which may be due to the chelation of RPP with Ca^2+^ to form a new substance with more stable structures. These results were similar to a study of white carp skin collagen peptide calcium chelates [30].

### 3.3. The Chelating Mechanism of RPP-Ca

#### 3.3.1. The Effect of Amino Acid Composition of RPP on Calcium Chelation Capacity

Given that the ability of RPP to enhance calcium absorption is intricately linked to the composition and concentration of amino acids, we utilized an amino acid analyzer to assess the prospective calcium-binding capacity of RPP. Table 3 indicates that the total concentration of amino acids having calcium-binding potential—namely aspartic acid, glutamic acid, histidine, threonine, cysteine, glycine, lysine, and serine—was 57.52 ± 0.08 g/100 g of protein peptides. This amount constitutes more than half of the total amino acid content, indicating that RPP possesses strong calcium-chelating capabilities. Notably, the levels of aspartic acid (15.23 ± 0.27 g/100 g chelate), glutamic acid (38.67 ± 0.28 g/100 g chelate), and histidine (3.74 ± 0.09 g/100 g chelate) in the RPP-Ca were significantly increased compared to RPP, demonstrating their enhanced calcium-binding activity (*p* < 0.05) [31]. In RPP, the content of hydrophobic amino acids was 32.40 ± 0.15 g/100 g protein peptide, which decreased to 18.53 ± 0.51 g/100 g chelate after chelation with Ca^2+^, indicating that the peptide–calcium chelation reaction may mainly occur on the surface of hydrophilic groups. These findings suggest that hydrophilic amino acids, such as histidine, aspartic acid, and glutamate, may serve as primary chelating sites for RPP and Ca^2+^, facilitating additional Ca^2+^ binding through electrostatic or coordination interactions [32].

#### 3.3.2. UV-Vis Spectroscopy Reveals the Interaction Sites Between Rice Protein Peptides and Calcium

UV spectroscopy is a widely used technique for analyzing the structural characteristics of chemical substances. It is well-established that when metal ions form complexes with organic ligands, new absorption peaks may appear, or existing peaks may shift or disappear [12]. At 197 nm, the greatest absorption peak for unchelated RPP was detected, which is consistent with the distinctive absorption of amide, carboxyl, and carbonyl bonds in bioactive peptides [33]. But following RPP’s chelation with Ca^2+^, the intensity dropped from 3.683 to 1.641, and the absorption peak moved to 194 nm. This alteration is probably attributable to the establishment of ligand interactions between Ca^2+^ and the amino nitrogen and carbonyl oxygen atoms in RPP [34]. In addition, phenylalanine absorption may be responsible for the RPP-Ca absorption peak at 259 nm [19]. In line with our findings, a similar blue shift in the maximum absorbance peak was observed in zeatin peptide when chelated with Ca^2+^ [32]. These results suggest that Ca^2+^ interacts with RPP to form a new compound.

#### 3.3.3. FTIR Spectroscopy Identified Key Functional Groups of Rice Protein Peptides Binding with Calcium

The FTIR of RPP and RPP-Ca are shown in Figure 1J. RPP showed high-frequency absorption at 3345.5 cm^−1^, which might be the absorption peak caused by the stretching vibration of the N-H bond [12]. After binding with Ca^2+^, the absorption peak red-shifted to 3363.5 cm^−1^, which may be caused by the enhancement of the electron cloud density of N-H due to the induction effect or dipole field effect, indicating that the amino group in the peptide chain may participate in chelating with Ca^2+^ [34]. After chelation with Ca^2+^, the absorption peak in RPP caused by the COO-symmetry vibration at 3074 cm^−1^ disappeared. At 1658.5 cm^−1^ and 1559 cm^−1^, RPP showed the amide-I band absorption peak caused by the stretching vibration of the C=O bond and the amide-II band absorption peak caused by the stretching vibration of the C-N bond and the bending vibration of the N-H bond, respectively [30]; while, they were blue-shifted to 1651 cm^−1^ and red-shifted to 1596.5 cm^−1^ in RPP-Ca, respectively, indicating that the C=O bond and the C-N bond may be involved in the binding of Ca^2+^. The redshift (from 1080 cm^−1^ to 1102.5 cm^−1^) and blue shift (from 628 cm^−1^ to 577.5 cm^−1^) of the absorption peaks due to the binding of calcium may be caused by C-O bond stretching vibration and N-H stretching vibration, respectively. These results suggested that Ca^2+^ may be chelated by carboxyl and amino groups on RPP to form RPP-Ca, which was consistent with a study on pig bone collagen peptide–calcium chelate [12].

### 3.4. Calcium Absorption-Promoting Effects and Mechanisms of RPP-Ca In Vitro

#### 3.4.1. Caco-2 Cell Monolayer Model and the Cytotoxicity of Peptide Calcium Chelates

**Figure 2:**
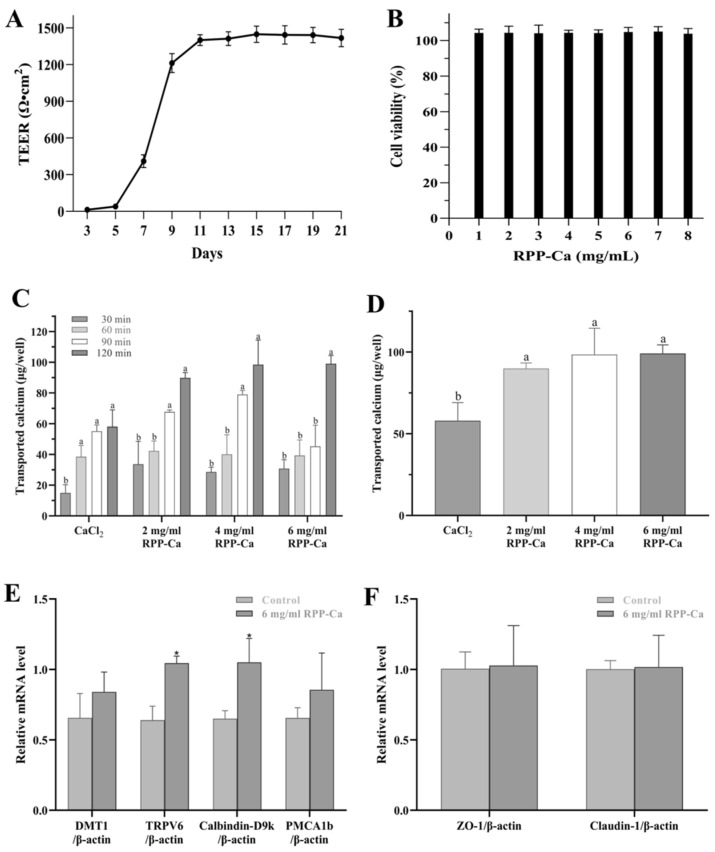
Effects of RPP-Ca on calcium transport and gene expression in Caco-2 cell monolayers. (**A**) Changes in transepithelial electrical resistance (TEER) of Caco-2 cell monolayers over 21 days of. (**B**) Effects of RPP-Ca (1–8 mg/mL) on Caco-2 cell viability. (**C**) Calcium transport across Caco-2 cell monolayers treated with CaCl_2_ or RPP-Ca (2, 4, and 6 mg/mL) for 30, 60, 90, and 120 min. (**D**) Total transported calcium across Caco-2 cell monolayers after 120 min of incubation. (**E**) Relative mRNA expression levels of calcium transport-related genes (DMT1, TRPV6, Calbindin-D9k, and PMCA1b). (**F**) Relative mRNA expression levels of tight junction-related genes (ZO-1 and Claudin-1). Different lowercase letters indicate significant differences (*p* < 0.05). * *p* < 0.05 compared with the control group.

To assess the tightness and integrity of Caco-2 cell monolayers, TEER as well as Papp values were determined. The TEER value increased slowly in 3–5 days (Figure 2A). The TEER values increased rapidly from 5 to 9 days due to the continuous division of Caco-2 cells and stabilized from day 15, indicating the formation of structured and integrated monolayers of Caco-2 cells [23]. The permeability test results of sodium fluorescein in the Caco-2 cell monolayer membrane after 21 days of culture are shown in Table 4. The Papp values of sodium fluorescein translocation in the Caco-2 cell monolayer for 0.5, 1, 1.5, and 2 h were 2.68 ± 0.25 × 10^−6^, 3.07 ± 0.47 × 10^−6^, 3.11 ± 0.43 × 10^−6^ and 3.32 ± 0.25 × 10^−6^ cm/s, respectively, indicating that the Caco-2 cells were closely packed in the monolayer membrane [24]. These results show that Caco-2 cells have established a monolayer membrane suitable for the subsequent calcium ion transport tests.

The impact of RPP-Ca at various concentrations (1–8 mg/mL) on the viability of Caco-2 cells was assessed using the MTT test. The survival percentage of Caco-2 cells remained around 100% following treatment with various concentrations of RPP-Ca samples for 24 h (Figure 2B), which indicated that these concentrations of RPP-Ca had no poisonous influence on Caco-2 cells. According to the above results, 2, 4, and 6 mg/mL were used for calcium absorption experiments.

#### 3.4.2. Effect of RPP-Ca on Ca^2+^ Absorption

The calcium transfer of 2, 4, and 6 mg/mL RPP-Ca at 30, 60, 90, and 120 min was assessed utilizing the Caco-2 cell monolayer model. 1.69 mM CaCl_2_ was used as a control, which was consistent with the calcium content in 2 mg/mL RPP-Ca. As shown in Figure 2C, calcium transport in each group increased with the prolongation of culture time. When cultured to 120 min, the calcium transport of RPP-Ca at 2, 4, and 6 mg/mL was 89.86 ± 2.42, 98.42 ± 11.36 and 99.04 ± 3.76 μg/well, respectively, compared with the CaCl_2_ control group (57.98 ± 7.78 μg/well), which indicated that the RPP-Ca chelate can significantly promote the transport of (Figure 2D, *p* < 0.05). Numerous investigations have demonstrated that in the Caco-2 cell monolayer, peptide–calcium chelates absorb Ca^2+^ more efficiently than inorganic calcium [32,35].

Glutamate and aspartate were associated with increased calcium uptake, and the positively charged basic amino acids (lysine, histidine, and arginine) could interact with cell membranes to facilitate the uptake and transport of Ca^2+^ [36]. Meanwhile, the histidine, aspartic, and glutamic acid content in RPP was raised after chelation with Ca^2+^. These findings suggested that RPP-Ca chelates promoted calcium transport might be related to aspartic acid, glutamic acid, and histidine.

Interestingly, the calcium ion transport did not differ significantly (*p* > 0.05) between the 2 mg/mL RPP-Ca group and the high-concentration RPP-Ca groups (4 mg/mL and 6 mg/mL). The attainment of the plateau may result from saturated calcium ion concentrations in Caco-2 cell monolayers, suggesting that the paracellular pathway may not be the main channel for calcium ion transport. Related previous research also discovered that desalted duck egg protein peptides facilitate calcium transfer independently of the paracellular pathway [37].

Overall, these results suggest that RPP-Ca significantly enhances calcium transport compared to inorganic calcium, potentially due to the Ca^2+^-binding properties of aspartate, glutamate, and histidine. Furthermore, RPP-Ca does not appear to rely on the paracellular pathway in the Caco-2 monolayer; however, whether it depends on other pathways requires further investigation.

#### 3.4.3. Effects of RPP-Ca on the Expression of Calcium Channel-Related Genes in Caco-2 Cells

In order to identify the precise mechanism by which RPP-Ca facilitates calcium transport, we examined the expression of relevant transporter markers. The uptake of dietary Ca^2+^ in the intestinal tract mostly involves passive and unsaturated paracellular transport, as well as active and saturable transcellular transport [38]. The relative expression levels of DMT1, transient receptor potential TRPV6, Calbindin-D9k, and PMCA1b genes in Caco-2 cells are shown in Figure 2E. In comparison to the control group, 6 mg/mL RPP-Ca significantly enhanced the relative expression of TRPV6 and Calbindin-D9k genes in Caco-2 cells (*p* < 0.05), with increases of 1.63 and 1.62 times that of the control group, respectively; concurrently, the relative expression of DMT1 and PMCA1b genes also increased, although the difference was not statistically significant (*p* > 0.05). TRPV6 in the transcellular pathway is a highly selective Ca^2+^ channel in Caco-2 cells, which exerts a vital effect on intestinal Ca^2+^ absorption [39]. Ca^2+^ enters intestinal epithelial cells via TRPV6, binds to Calbindin-D9k, and is then pumped out of cells by PMCA1b [40]. Casein phosphopeptide promoted calcium uptake by interacting with TRPV6 in Caco-2 cells [41]. Duck egg white peptide was also found to promote calcium absorption by upregulating TRPV6 and Calbindin-D9k in Caco-2 cells [42]. Intestinal tight junctions (TJs) are closely related to paracellular transport, and Ca^2+^ can be transported through TJs (such as ZO-1 and claudin-1) [43,44]. To further confirm whether the calcium uptake effect of RPP-Ca is not dependent on the paracellular pathway, the relative expression levels of ZO-1 and claudin-1 genes were measured. Figure 2F illustrates that following RPP-Ca therapy, there were no significant variations in the relative expression levels of the ZO-1 and claudin-1 genes as compared to the control group (*p* > 0.05). In conclusion, RPP-Ca facilitated calcium absorption via the transcellular pathway by enhancing the expression of TRPV6 and Calbindin-D9k. Although mRNA upregulation serves as an intermediate signal rather than directly demonstrating protein synthesis, our parallel study on specific active peptide components (AHVGMSGEEPE-calcium chelator) has confirmed through Western blot that TRPV6 and Calbindin-D9k are efficiently translated at the protein level [21]. This supporting evidence supports the following premise: the transcriptional activation triggered by a large amount of RPP-Ca can successfully cascade to the functional expression of transport proteins.

### 3.5. RPP-Ca Promotes Calcium Absorption and Bone Synthesis In Vivo

Although RPP-Ca has been observed to enhance calcium absorption significantly in Caco-2, its role in vivo has not been confirmed. Therefore, we looked for evidence of the impact of RPP-Ca on calcium uptake using animal experiments and compared its effect with that of inorganic calcium supplements. In this study, CaCl2 was selected as the primary reference control because its complete dissociation in solution provides an absolute baseline of free Ca^2+^ ions, allowing for a precise evaluation of the biological advantage conferred specifically by peptide chelation.

#### 3.5.1. RPP-Ca Alleviates the Imbalance of Bone Metabolism Caused by a Calcium-F Diet

**Figure 3:**
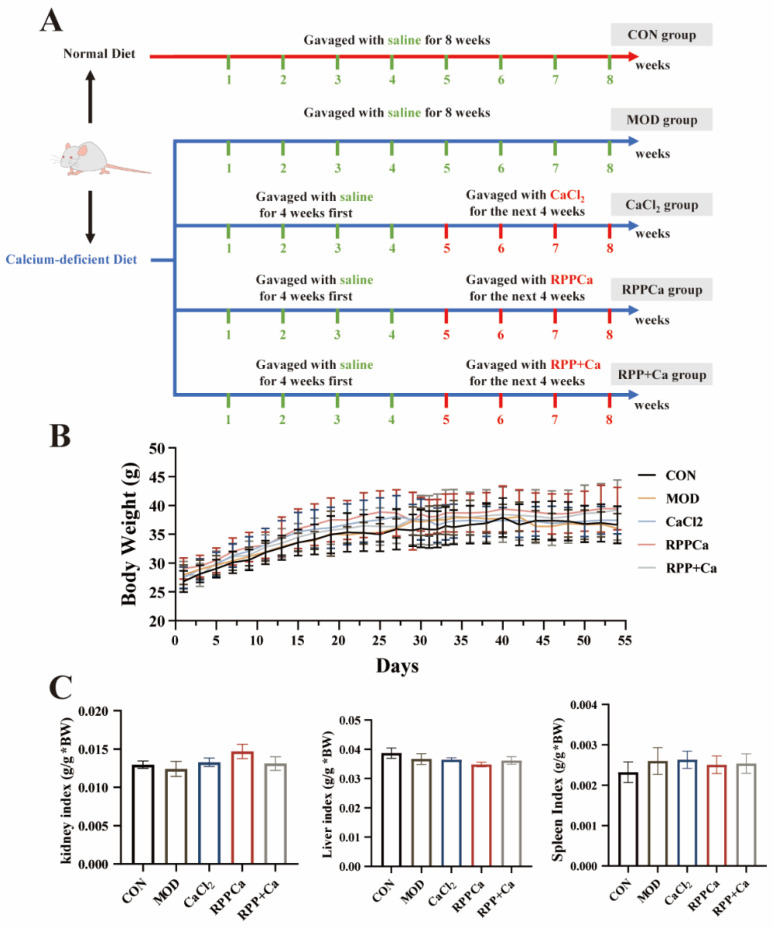
Experimental design and effects of RPP-Ca on body weight and organ indices in calcium-deficient mice. (**A**) Schematic timeline of the animal experimental design and grouping over 8 weeks. (**B**) Changes in rat body weight during the 55-day experimental period. (**C**) Kidney, liver, and spleen indices of mice in different experimental groups at the end of the study. The * symbol in the picture represents a multiplication sign.

**Figure 4:**
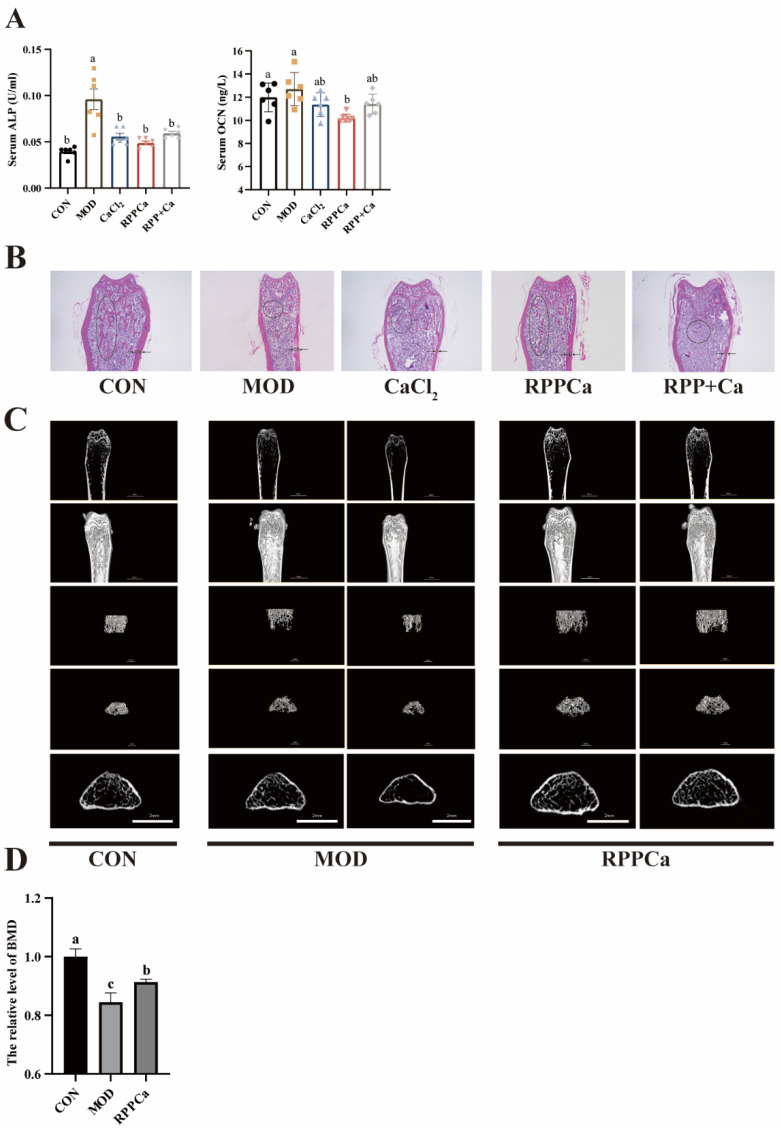
Effects of RPP-Ca on serum bone biomarkers and bone histomorphology in calcium-deficient mice. (**A**) Serum levels of alkaline phosphatase (ALP) and osteocalcin (OCN). (**B**) Hematoxylin and eosin (H&E) staining of rat femur tissue sections (scale bars or structures indicated by arrows/circles). (**C**) Micro-CT images showing three-dimensional trabecular bone microarchitecture of the femur. (**D**) Relative level of bone mineral density (BMD) among control (CON), model (MOD), and RPPCa groups. Bars marked by different superscript letters (a, b, c) differ significantly (*p* < 0.05).

Figure 3B illustrates that the body weight of each group was comparable, signifying similar growth rates among all groups. The organ index was subsequently assessed to determine whether RPP-Ca exhibited any side effects. Figure 3C illustrates that there was no statistically significant difference in organ index between the RPP-Ca and control (CON) groups (*p* > 0.05), indicating that RPP-Ca did not negatively impact body growth and general health. Additionally, we measured serum levels of alkaline phosphatase (ALP) and osteocalcin (OCN), which serve as classic biochemical markers of bone turnover. The MOD group’s levels of ALP and OCN were much greater than those of the CON group, as shown in Figure 4A. This suggests that the imbalance in bone metabolism caused an enhanced rate of bone turnover, which validates that the calcium deficiency model was established successfully. However, in the calcium supplementation groups (CaCl_2_, RPP-Ca, and RPP + Ca), a notable downward trend in ALP levels was observed, demonstrating that calcium supplementation effectively reversed the calcium metabolism imbalance. Notably, OCN levels were markedly downregulated among the RPP-Ca group compared to the inorganic calcium supplement group, indicating that RPP-Ca was more efficacious in recovering calcium metabolism. Previous studies [45,46] have demonstrated that individuals with osteoporosis exhibit imbalances in bone loss and bone growth. Enhanced bone resorption promotes the growth of bones, resulting in higher levels of bone formation indicators, such as OCN and ALP, in the bloodstream. Thus, the significant decrease in ALP and OCN levels suggests that calcium intake can reverse calcium metabolic imbalances and that RPP-Ca is more effective than inorganic calcium in reversing OCN imbalances. However, whether the reversal of bone metabolism further enhances bone structure and bone strength has not been proven.

#### 3.5.2. Effects of RPP-Ca on Bone Microstructure

To further explain the effect of ALP and OCN changes on bone synthesis, we used H&E and Micro-computed tomography (Micro-CT) to evaluate changes in bone microstructure. H&E staining was employed to examine the impact of various calcium supplements on the microstructure of bone trabeculae. The trabecular structure in the CON group exhibited a compact structure, uniform thickness, and good continuity (Figure 4B). In contrast, the MOD group displayed sparse and unevenly arranged trabecular bone, with a significantly reduced coverage area. After calcium supplementation, particularly in the RPP-Ca group, there was a notable improvement in both the density and thickness of the trabeculae. Furthermore, when examining cortical bone (CB), the CON group showed a thick and dense layer, while the MOD group showed a thinner layer. However, this phenomenon was significantly reversed in the RPP-Ca group, and the thickness and density of the CB recovered. These results indicate that RPP-Ca supplementation is more effective than inorganic calcium supplementation in reversing the damage to bone microstructure in mice with osteoporosis caused by calcium deficiency.

Additionally, we reconstructed the bone structure of the CON, MOD, and RPP-Ca groups using micro-CT, providing a more detailed and comprehensive view of the trabecular microstructure compared to H&E staining. Figure 4C illustrates that the trabecular bone in the CON group exhibited a well-organized structure. Conversely, the MOD group had a notable decrease in the thickness of the outer cortical ring, accompanied by a sparse distribution of the inner trabeculae. In the RPP-Ca group, both the thickness of the outer cortical ring and the density of the inner trabeculae were dramatically elevated, resulting in a decreased gap between trabecular structures. Prior studies have shown that a 50% drop in dietary calcium results in diminished bone strength and mineral density [47]. Consequently, we employed bone mineral density (BMD) as a diagnostic indicator for osteoporosis. This study revealed similar results in the MOD group (Figure 4D), where the relative BMD was significantly lower (0.8449 ± 0.01559) compared to the CON group (1.000 ± 0.01543). After RPP-Ca administration, the BMD significantly increased to 0.9131 ± 0.01038. These findings indicate that RPP-Ca can significantly ameliorate the structural damage to bone resulting from osteoporosis and enhance bone density.

#### 3.5.3. Improvement of Bone Biomechanical Indexes by RPP-Ca

**Figure 5:**
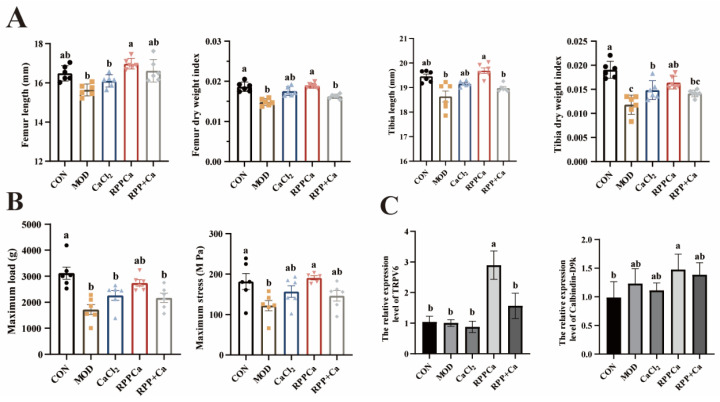
Effects of RPP-Ca on bone physical properties, biomechanical parameters, and intestinal gene expressions in calcium-deficient mice. (**A**) Femur length, femur dry weight index, tibia length, and tibia dry weight index of mice. (**B**) Bone biomechanical properties including maximum load and maximum stress of the femur. (**C**) Relative mRNA expression levels of TRPV6 and Calbindin-D9k in the duodenum or small intestine of mice. Bars marked by different superscript letters (a, b, c) differ significantly (*p* < 0.05).

Having identified structural changes in the femur, we further examined the associated biomechanical changes in bone. Figure 5A illustrates that, in comparison to the RPP-Ca group, bone strength was markedly improved, with both the maximum load (2741 ± 118.1 g) and stress (190.7 ± 5.15 MPa) of the femur showing notable increases. In contrast, the MOD group exhibited significantly reduced maximum load (1721 ± 187.5 g) and stress (122.0 ± 12.65 MPa) values. Notably, the improvement in bone strength in the RPP-Ca group was even greater than that observed in the groups supplemented with inorganic calcium. Additionally, femur length and the dry weight index were both increased in the RPP-Ca group (Figure 5B), and these values were markedly elevated compared to those in the MOD and inorganic calcium supplementation groups. Li et al. [48] reported similar findings, noting improvements in maximum load and maximum deflection in the puerarin-treated group, which alleviated OVX-induced osteoporosis. The above experimental results confirmed that RPP-Ca could significantly improve the biomechanical quality of bone and increase the load-bearing capabilities of bone, exhibiting superior efficacy compared to both the CaCl_2_ group and the RPP + Ca group. However, the underlying mechanisms of these improvements remain unclear.

#### 3.5.4. Validation of Calcium Absorption Pathway

To determine whether the regulatory mechanisms of RPP-Ca in vivo are consistent with those we previously validated in Caco-2 cells, we used qPCR to detect the relative gene expression levels of TRPV6 and calbindin-d9k in colon tissue. As we expected (Figure 5C), TRPV6 and calbindin-d9k were upregulated in the RPP-Ca group, but inorganic calcium also increased calbindin-d9k levels, possibly because dietary calcium stimulated calcium 9k [49]. Yang et al. [50] discovered that calcium soy peptide enhances calcium absorption by elevating the expression of TRPV6 and calbindin-d9k in the kidneys. Hou et al. [42] discovered that peptides derived from desalted duck egg white enhanced the expression of TRPV6 and calbindin-d9k in the colon, hence improving calcium absorption.

Overall, RPP-Ca upregulates TRPV6 and calcium-binding protein-D9K, enhancing calcium transport in the small intestine, and thereby promoting calcium utilization and bone formation.

## 4. Conclusions

In this study, we identified RPP-Ca that promotes calcium absorption and bone synthesis, optimized its synthesis conditions, and elucidated its synthesis mechanism.

During the process of synthesizing RPPCa, we found that the calcium binding ability was influenced by several factors, including pH, reaction time, peptide-to-calcium mass ratio, and reaction temperature. Optimal conditions identified through orthogonal optimization were pH 10, a reaction time of 50 min, a peptide-to-calcium mass ratio of 2:1, and a reaction temperature of 70 °C, resulting in a calcium binding capacity of 93.98 ± 1.99 mg/g chelate. We further measured the stability of this chelated complex compared with the pure peptide. Scanning electron microscopy (SEM) revealed a transformation of the RPP surface from a smooth porous structure to a dense granular structure post-chelation with Ca^2+^. DSC and TGA indicated that the thermal stability of RPP-Ca was enhanced following chelation.

To understand the chelation mechanism of RPP-Ca, we analyzed the amino acid composition of RPP and identified that aspartic acid (Asp), glutamic acid (Glu), and histidine (His) play crucial roles in the interaction between RPP and calcium. UV–visible and FTIR spectroscopy analyses also suggested that these three amino acids likely chelate with calcium through their carboxyl and amino functional groups.

After elucidating the synthesis and chelation properties of RPP-Ca, we conducted in vitro and in vivo experiments to explore its role in promoting calcium absorption. In the Caco-2 cell model, calcium transport for RPP-Ca at concentrations of 2, 4, and 6 mg/mL was measured at 89.86 ± 2.42, 98.42 ± 11.36, and 99.04 ± 3.76 µg/well, respectively, significantly (*p* < 0.05) exceeding the control group (57.98 ± 7.78 µg/well). Additionally, RPP-Ca demonstrated superior absorbability compared to inorganic calcium and facilitated calcium transport by upregulating TRPV6 and Calbindin-D9k gene expression, independent of paracellular pathways. In a calcium-deficient dietary model, RPP-Ca, unlike inorganic calcium, significantly increased the elevated bone turnover rate, stimulated new bone formation, and improved calcium absorption in the small intestine, thereby enhancing calcium bioavailability. Moreover, RPP-Ca effectively improved the biomechanical properties and microstructure of the femur and tibia. Consistent with findings in Caco-2 cells, the expression of TRPV6 and Calbindin-D9k genes was found to be elevated in the colon of the RPP-Ca group, indicating that RPP-Ca may effectively enhance calcium transport. Furthermore, although the current research results indicate that RPP-Ca has significant bone formation benefits in the mouse model, a comprehensive study on its stability during storage, long-term toxicological safety, and absolute systemic bioavailability remains a key task for the next step. It is crucial that, since preclinical models cannot fully simulate human metabolic kinetics, strict human clinical trials must be conducted before determining the actual nutritional efficacy and commercial application of RPP-Ca as a functional enhancer.

The results of this study indicate that industrially produced rice protein peptides have great potential as calcium supplementation raw materials. The functional development of RPP-Ca can not only increase the value of broken rice, expand the application scope of RPP, but also enrich the types of calcium supplementation. However, before practical application, extensive clinical research is necessary.

## Figures and Tables

**Table 1:** Factors and level design of orthogonal experiment.

Level	Factors			
	A: The Ratio of Peptide: Calcium	B: pH	C: Time (Min)	D: Temperature (°C)
1	1:1	8	40	50
2	2:1	9	50	60
3	3:1	10	60	70

**Table 2:** Results of orthogonal experiment on preparation of RPP-Ca.

Test No.	A	B	C	D	Calcium Chelating Capacity (mg/g Chelate)
	The Ratio of Peptide: Calcium	pH	Time	Temperature	
	1 (1:1)	1 (8)	1 (40 min)	1 (50 °C)	77.22
	1 (1:1)	2 (9)	2 (50 min)	2 (60 °C)	90.63
	1 (1:1)	3 (10)	3 (60 min)	3 (70 °C)	92.27
	2 (2:1)	1 (8)	2 (50 min)	3 (70 °C)	82.27
	2 (2:1)	2 (9)	3 (60 min)	1 (50 °C)	87.84
	2 (2:1)	3 (10)	1 (40 min)	2 (60 °C)	90.48
	3 (3:1)	1 (8)	3 (60 min)	2 (60 °C)	71.68
	3 (3:1)	2 (9)	1 (40 min)	3 (70 °C)	80.20
	3 (3:1)	3 (10)	2 (50 min)	1 (50 °C)	89.35
K1	260.1147	231.1661	247.8980	254.4108	
K2	265.1666	258.6681	262.2489	252.7808	
K3	262.3803	272.1007	251.7880	254.7433	
k1	86.7049	77.0554	82.6327	84.8036	
k2	88.3889	86.2227	87.4163	84.2603	
k3	87.4601	90.7002	83.9293	84.9144	
R	1.6840	13.6449	4.7836	0.6541	

**Table 3:** Amino acid composition of RPP-Ca.

Amino Acid	RPP ^A^ (g/100 g)	RPP-Ca (g/100 g)
Asp	11.33 ± 0.01	15.23 ± 0.27
Thr	3.45 ± 0.01	3.04 ± 0.10
Ser	5.90 ± 0.00	5.31 ± 0.04
Glu	23.60 ± 0.01	38.67 ± 0.28
Gly	4.88 ± 0.01	4.26 ± 0.05
Ala	6.47 ± 0.01	3.74 ± 0.15
Cys	0.72 ± 0.03	ND
Val	5.29 ± 0.01	3.46 ± 0.07
Met	1.33 ± 0.06	0.98 ± 0.08
Ile	3.07 ± 0.02	1.69 ± 0.07
Leu	6.09 ± 0.02	3.01 ± 0.04
Tyr	3.72 ± 0.05	3.66 ± 0.11
Phe	3.14 ± 0.01	2.37 ± 0.13
Lys	4.75 ± 0.00	4.02 ± 0.02
His	2.89 ± 0.01	3.74 ± 0.09
Arg	9.91 ± 0.05	6.80 ± 0.08
Pro	3.46 ± 0.07	ND
EAA	27.13 ± 0.06	18.58 ± 0.42
NEAA	72.87 ± 0.06	81.42 ± 0.42

^A^ Data from our previously published article [5]; ND: not detected.

**Table 4:** Apparent permeability coefficient (Papp) of sodium fluorescein in Caco-2 Monolayer.

Time (h)	Papp (×10^−6^ cm/s)
0.5	2.68 ± 0.25
1	3.07 ± 0.47
1.5	3.11 ± 0.43
2	3.32 ± 0.25

## Data Availability

The original contributions presented in this study are included in the article/Supplementary Material. Further inquiries can be directed to the corresponding authors.

## Supplementary Materials

The following supporting information can be downloaded at https://www.mdpi.com/article/10.3390/foods15142490/s1: Table S1. Primer sequence of genes in RT-qPCR. Table S2. The scan conditions of Micro-CT.
