# Supplementary material for: From Synthesis Optimization to Chelation Mechanism: A Rice Protein Peptide–Calcium Complex Enhances Intestinal Calcium Absorption and Bone Formation via the TRPV6-Calbindin9k Axis
Source: Foods. 2026 Jul 14;15(14):2490. doi: 10.3390/foods15142490 (PMC13409443; doi:10.3390/foods15142490)
Supplement: Supplementary file 1 [file foods-15-02490-s001.zip › foods-4389868-supplementary.pdf]

Support Information

From Synthesis Optimization to Chelation Mechanism: A Rice Protein Peptide-Calcium Complex Enhances Intestinal Calcium Absorption and Bone Formation via the TRPV6-Calbindin9k Axis

Yue Tian[a,b,1], Wenting Yang[a, 1], Yangzheng He[a], Xin Bi [a,b,*], Yong Sun[a,*]

[a] State Key Laboratory of Food Science and Resources, Nanchang University, Nanchang 330047, Jiangxi, China

[b] Jiangxi Medicine Academy of Nutrition and Health Management, Nanchang University, Nanchang 330006, Jiangxi, China

Content

1. **Primer sequence of genes in RT-PCR.** 3

2. **The scan conditions of Micro-CT** 4

1. Primer sequence of genes in RT-PCR.

Table S1. Primer sequence of genes in RT-qPCR.

Gene	Forward（5’-3’）	Reverse（5’-3’）
β-actin	ATCACTATTGGCAACGAGCG	TCAGCAATGCCTGGGTACAT
DMT1	GTGGAGTTGGCTATCATCGGC	GCTCTGGCTGGGTTTCACTGTA
TRPV6	CACCCAGTGGACGTATGGAC	CTCGTGCGGTTATTGGTCCT
Calbindin-D9k	GGCAACCAGACACCAGAATGA	TGACAACTGGTCTGGATCACC
PMCA1b	AGTGATTGTTGCTTTTACGGGC	AGAGACTCAGTGGGTGGTTCCG
ZO-1	TGAGGCAGCTCACATAATGC	GGTCTCTGCTGGCTTGTTTC
Claudin-1	TGGTCAGGCTCTCTTCACTG	TTGGATAGGGCCTTGGTGTT

2. The scan conditions of Micro-CT

Table S2. The scan conditions of Micro-CT.

Steps	Descriptions
1.Scanning	The sample was removed from the fixative, the excess liquid was dried with gauze, the sample was placed in the scanning bed of the instrument and then started to be scanned. The scanning parameter was set to the pixel size is 0.026*0.026*0.026 mm and the original image was obtained after scanning.
2.Reconstruction	Reconstruct the original image using the 3D reconstruction software Recon. The reconstruction method is FDK which to start the image reconstruction.
3.Analysis	Use the data analysis software Avatar to analyze the ROl of the target area, select the same area for analysis for all samples. The threshold assigned to all samples was 1140 hounsfield unit to analyze and export the data.
